# Using multiple traits to assess the potential of introduced and native vines to proliferate in a tropical region

**DOI:** 10.1002/ece3.2588

**Published:** 2016-11-21

**Authors:** Diana L. Delgado, Josimar Figueroa, Carla Restrepo

**Affiliations:** ^1^ Department of BiologyUniversity of Puerto Rico‐Río PiedrasSan JuanPuerto Rico

**Keywords:** alien species, climbing plants, invasive vines, plant traits, proliferating vines

## Abstract

Predicting the invasive potential of introduced species remains an ongoing challenge due to the multiple interacting regional and global processes that facilitate the introduction and proliferation of alien species. This may be particularly true in regions where native species are increasingly reported as expanding and impacting ecosystems in ways indistinguishable from alien ones. Current approaches to assess the invasive potential of plants may be limited by the choice of traits used and the exclusion of native species. To overcome these limitations, we develop a novel approach that focuses on all species—native and alien—within a functional group of plants to predict their proliferation status. Our approach relied on the development of an extensive database of extrinsic and intrinsic traits for Puerto Rican vines with the goal of generating a predictive model of vine proliferation status. We test three hypotheses linking origin, extrinsic and intrinsic traits, and proliferation status. We found that the origin of proliferating vines was associated with only one out of seven traits, namely plant use. We also found that proliferation status was associated with all but two traits, namely life span and climbing mechanism. Finally, a classification tree analysis identified five variables as good predictors of proliferation status and used them to split the species into six groups characterized by a unique suite of traits, three of them included proliferating species. The development of tools to identify potential proliferating species is critical for management and conservation purposes. Tools that can minimize biases and make predictions based on trait data easily obtainable are particularly needed in regions with a high taxonomic and functional diversity, and with limited ecological knowledge of individual species. In addition, these tools should be capable of incorporating native species since an increasing number of native species are behaving like invasive aliens.

## 1 Introduction

Predicting the invasive potential of introduced plant species remains an ongoing challenge. First, globalization facilitates the movement of species at scales rarely seen before (Meyerson & Mooney, 2007; Perrings, Mooney, & Williamson, 2010). Second, boom‐and‐bust cycles in the production of agricultural goods contribute to spatial and temporal shifts in the establishment of alien species (Clough, Faust, & Tscharnetke, 2009; Ha & Shively, 2008). Third, the aforementioned shifts are often part of major socioeconomic transformations that may be followed by de‐intensification and abandonment of agriculture (Ladwig & Meiners, 2009), including the creation of suitable conditions for the spread of alien species (Grau et al., 2008; Johnson, Litvaitis, Lee, & Frey, 2006; Standish, Cramer, & Hobbs, 2008). Lastly, global changes may have the potential to interact with a variety of socioeconomic processes facilitating the proliferation of alien as well as native species (Bellard et al., 2013; Seebens et al., 2015). In fact, many native species are increasingly reported as expanding and impacting ecosystems in ways indistinguishable from alien species (Carey, Sanderson, Barnas, & Olden, 2012; Simberloff, 2011). There is no sign that the above processes will ameliorate in years to come, and thus the development of tools aimed at identifying alien species with a potential to proliferate and to monitor species already introduced is critical for management and conservation purposes (Essl et al., 2015).

Three approaches based on plant traits have been useful to assess the potential of introduced species to become invasive. One approach focuses on *individual plant species* and combines intrinsic traits (e.g., propagule availability, dispersal, and local abundance) with biophysical variables (e.g., temperature, soil moisture, forest cover) to model the spread of introduced species (Albright, Anderson, Keuler, Pearson, & Turner, 2009; Higgins, Richardson, & Cowling, 1996; Ibáñez et al., 2014). The inclusion of intrinsic trait data representing growth and dispersal processes adds realism to the models, but for diverse and little known plant assemblages, these data may be difficult to obtain. Another approach focuses on *all alien species introduced to a given region* and integrates intrinsic (e.g., presence of undesirable traits, dispersal mechanisms, and reproduction) and extrinsic (history of introduction, biogeography) traits to provide a semiquantitative or quantitative assessment of species' invasion risk (Daehler & Carino, 2000; Huang, Wu, Bai, Zhou, & Wang, 2009; Pheloung, 1995; Pyšek, Křivánek, & Jarošík, 2009; Rojas‐Sandoval & Acevedo‐Rodriguez, 2015). Among the *undesirable traits* are those that contribute to plant noxiousness (e.g., toxic compounds) and persistence (e.g., shade tolerant), or that increase their potential to become an environmental weed (e.g., climbing habit) (Pheloung, 1995). The drawback of this approach is that species with undesirable traits may be predisposed to be classified as potential invaders even though this group can include noninvasive species of economic importance. The third and last approach focuses on *the subset of introduced species of a given functional or taxonomic group reported in a given region* and uses extrinsic traits (e.g., years since introduction, plant use, origin, and habitat) to predict plant spread (Harris, Murray, Hose, & Hamilton, 2007; Wilson et al., 2011). It has been shown that plant use is an extrinsic trait that is critical to understand the introduction and establishment phases of invasion in a given region. In particular, alien species of horticultural and economic importance are intentionally introduced and intensively sown, thus facilitating their proliferation (Dehnen‐Schmutz, Williamson, Touza, & Perrings, 2007; Reichard & White, 2001). Yet, native species may also proliferate and act similarly as alien invasive species (Kirkham, 2005; Knapp & Soulé, 1998; Taylor & Kumar, 2016).

The inherent limitations of the approaches discussed previously may introduce biases when assessing the potential of plants to invade or proliferate. A case in point is represented by vines, a group of herbaceous, climbing plants that are increasingly proliferating in fragmented habitats and areas undergoing agricultural de‐intensification and abandonment around the world. In these regions, invasive vines smother plant canopies, forest edges, and infrastructure alike over vast areas (Blaustein, 2001; Ernst & Ketner, 2007; Kirkham, 2005; Mackey et al., 1996). Vines have in common a climbing habit and a reliance on other plants to access resources—two of the traits that are considered undesirable in many risk assessment studies (Daehler & Carino, 2000; Pheloung, 1995; Rojas‐Sandoval & Acevedo‐Rodriguez, 2015). Yet, not all vine species are proliferating and not all proliferating vines are alien (e.g., Harris et al., 2007; Kirkham, 2005; Liengola, 2008; Taylor & Kumar, 2016). Thus, climbing habit and origin by themselves are not sufficient to explain the proliferation status of vine species in a variety of environments.

**Figure 1:**
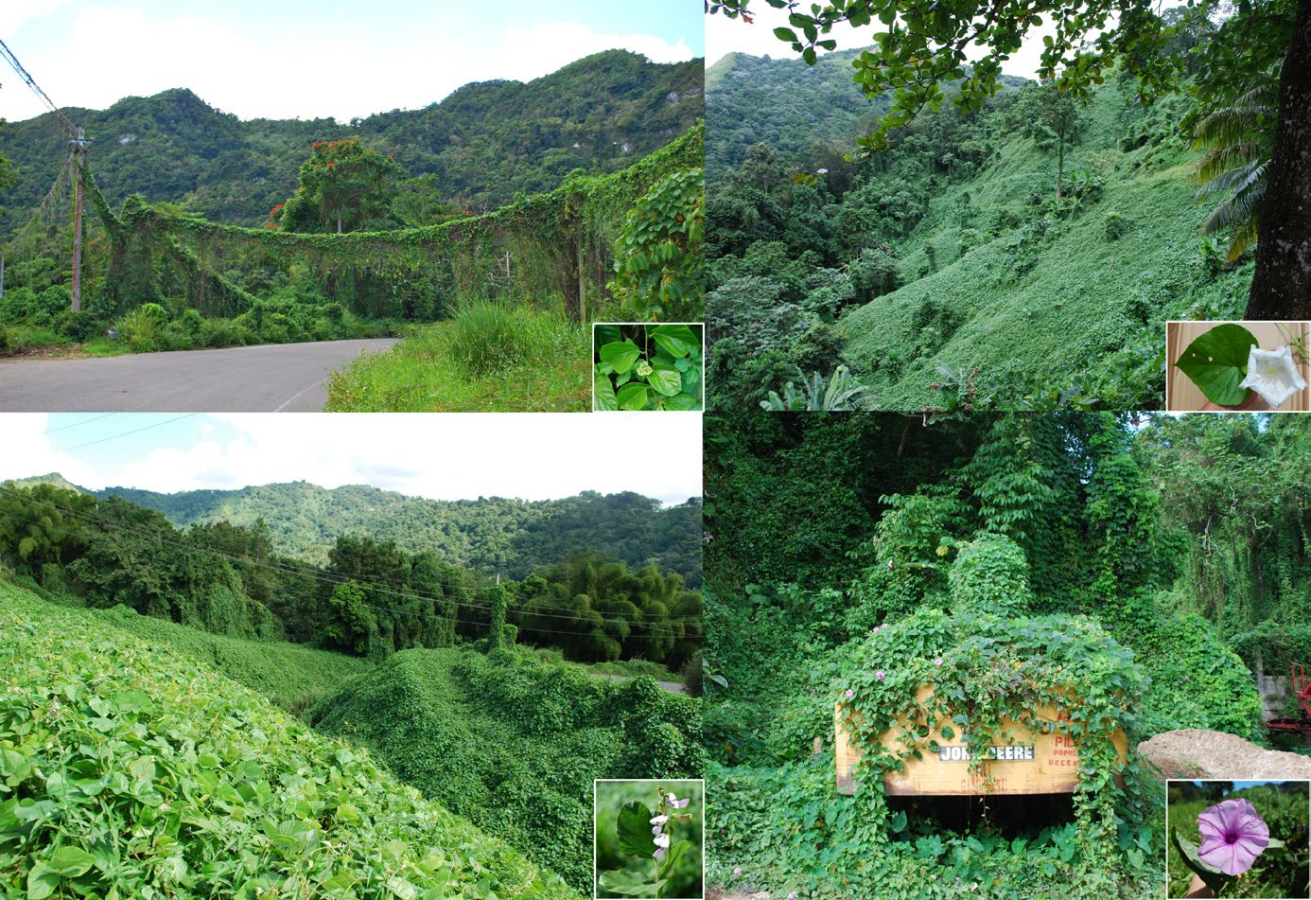
Examples of areas invaded by vines and the dominant vine species spreading in the area. Vine species from left to right in the upper panel: *Cissus verticillata* and *Ipomoea alba*. Lower panel: *Pueraria phaseoloides* and *Ipomoea tiliacea*

Here, we develop a novel approach that focuses on *all species—native and alien—within a functional group of plants* to predict their proliferation status while overcoming some of the limitations discussed previously. Our approach leverages the diversity of functional types present in vines, as well as the proliferation of alien and native vines alike. Vine diversity is manifested in their diverse climbing mechanisms and host preferences (Gentry, 1991; Hegarty & Caballé, 1991), life history strategies and patterns of biomass allocation (Gallagher & Leishman, 2012; Hairiah & van Noordwijk, 1989; Kolawole & Kang, 1997; Lambert & Arnason, 1986), and horticultural and economic value (Bovell‐Benjamin, 2007; Nicodemo et al., 2015; Ortiz‐Ceballos, Aguirre‐Rivera, Osorio‐Arce, & Peña‐Valdivia, 2012). Additionally, in numerous regions around the globe, including the island of Puerto Rico, a variety of vine species are smothering plant canopies, forest edges, and infrastructure alike over vast areas (Figure 1; Delgado, 2015; Kirkham, 2005; Taylor & Kumar, 2016).

Our approach relied on the development of an extensive database on extrinsic and intrinsic traits for vines reported in the island of Puerto Rico with the ultimate goal to generate a predictive model of vine proliferation status. We set to test three hypotheses linking origin, extrinsic and intrinsic traits, and proliferation status. Because both alien and native species are proliferating in the island, we hypothesized that irrespective of their origin, these two groups of vines shared similar traits. We further hypothesized that proliferating and nonproliferating vines were characterized by different traits. If we could establish an association between proliferation status and multiple traits, we further hypothesized that these traits could be used to predict vine proliferation status. The development of a model that can predict plant proliferation status could become an important monitoring tool, especially in diverse regions like Puerto Rico, for which knowledge of individual species is limited.

## 2 Methods

### 2.1 Study area

**Figure 2:**
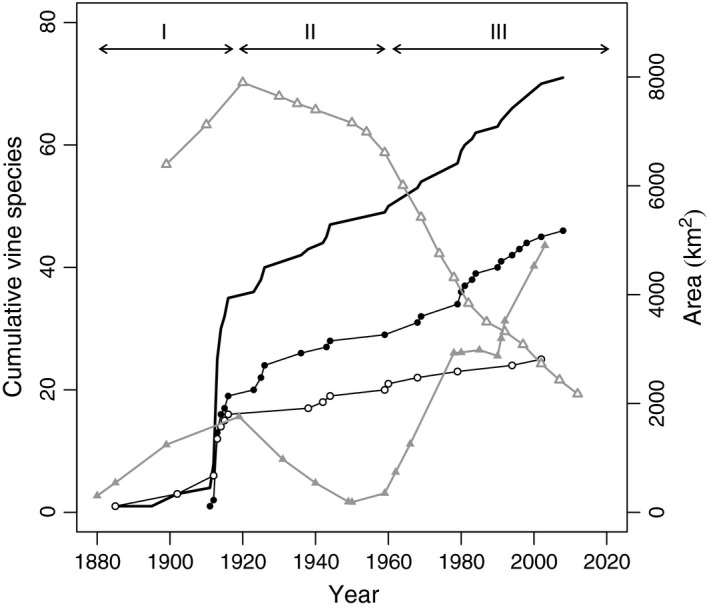
Land‐use change and the cumulative number of alien vines introduced to Puerto Rico over time. Area covered by farmland is represented by empty triangles (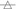) and forest area is represented with filled triangles (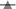). The cumulative number of proliferating alien species is represented with empty circles (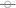) and the number of nonproliferating alien species is represented with filled circles (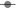). The solid black line represents the cumulative number of all introduced vine species. Changes in farmland area were used to define three periods of introductions: (1) 1880–1920 when the largest area in farmland area was recorded, (2) 1921–1960 when the area in farmlands started the decrease and forest area to increase, and (3) 1961–2008 when the area in farmland reached its lowest level and forest area increased to its highest levels (Appendix S1)

Puerto Rico, the smallest and easternmost island of the Greater Antilles, encompasses diverse life zones as well as land uses (Ewel & Whitmore, 1973; Helmer, Brandeis, Lugo, & Kennaway, 2008; Rosenberry, Gudmundson, & Samper, 1995). The island has undergone a well‐documented forest transition with a decline in forest cover associated with an expanding agricultural economy during the beginning of the 20th century (Figure 2). By the early 1960s, however, the decline in forest cover was reversed due to a transition from an agricultural to an industrial‐based economy (Yackulic et al., 2011; Figure 2; Appendix S1). Over the years, the dynamics of land‐use change coupled with the development of a dense network of public infrastructure have been important in creating diverse habitats amply used by native and alien vines (Acevedo‐Rodriguez, 2005; Delgado, 2015). Although vines proliferate both in urban and rural environments, it is in rural areas where their impact might be greatest. For example, in Delgado's (2015) study area in central Puerto Rico, vines cover ~48 km^2^ of a total area of 1,763 km^2^; in this region like in other rural areas of the Island, vines grow mainly on abandoned pastures, forest edges, and young secondary forests.

### 2.2 Construction of climbing plant databases

We compiled a list of Puerto Rican climbing plants based initially on Acevedo‐Rodriguez (2005) but subsequently updated it based on Axelrod (2011). For all climbing species, we obtained information on taxonomy, life form, origin, and proliferation status. All climbing species were first classified by taxonomic family and genera. Then, the species were classified by life form into lianas (woody climbing plants with thick stems growing in mature forests) and vines (nonwoody and subwoody climbing species, including shrubs, with thin stems often growing in disturbed areas and forest edges) (Gentry, 1991). Species were further classified by origin into alien (species introduced accidentally or intentionally as a result of human activity) or native (species whose historical distribution included Puerto Rico). Likewise, climbing species were classified by proliferation status into proliferating and nonproliferating species. Proliferating species include alien invasive (species producing large numbers of reproductive offspring and spreading into new areas) and native encroaching or weedy species (species increasing in density, cover, or biomass) (Carey et al., 2012; Richardson et al., 2000). In the field, these two sets of characteristics translate into vines' smothering behavior, that is., vines covering plant canopies, forest edges, and infrastructure alike over extensive areas. We used published accounts of weedy and invasive plants in Puerto Rico (Más & Lugo‐Torres, 2013), as well as our own observations (Delgado, 2015) and the opinion of an expert on Puerto Rican plants (E. Santiago, personal communication) to classify vines according to their proliferation status based on the definitions given above.

We focused next on the subset of climbing species classified as vines because they represent the vast majority of climbing plants and include most of the species that are proliferating in the island. We ran an extensive literature review and herbaria search to collect information on species' distribution, intrinsic and extrinsic traits, and proliferation status outside of Puerto Rico (Table 1; for the complete database, see Appendix S2). All the vine species in our database that were listed in World and Regional lists as invasive or weedy were considered as proliferating outside of Puerto Rico (Appendix S2).

**Table 1:** Vine traits included in this study with the corresponding sources of information

Trait class	Trait	Trait values	Source trait definition	Source trait data
Taxonomy and Distribution	Family	Family names		1, 2
	Genus	Genus names		1, 2
	Species	Species names		1, 2
	Origin	Native, Alien	9	1, 2
	Distribution*	Endemic, Native, Casual alien, Naturalized alien	9	1, 2, 12
	Historic biogeographic realms*	Afrotropics, Australasia, Indo‐Malay, Neotropics, Oceania, Palearctic	7	1, 2, 13
	Current biogeographic realms	Afrotropics, Australasia, Indo‐Malay, Nearctic, Neotropics, Oceania, Palearctic	7	12, 13
	Time of earliest collection	Year		14
	Local abundance*	Common, Uncommon, Rare	17	2
Proliferation status	Proliferating in Puerto Rico	Yes, No	3, 9, 17	6, 15, 16
	Invasive or weedy outside of Puerto Rico	Yes, No	9	18
Intrinsic	Life form	Vine, Liana	4	1, 2
	Life span*	Annual, Perennial, Annual/Perennial	5	12
	Climbing mechanism*	*Active*: Aerial Roots, Tendrils, Twining; *Passive*: Sarmentose, Scandent, Spines	1, 8	1
	Fruit type – general*	Simple, Compound, Rhexocarpic, Schizocarpic	10	1, 11, 18
	Fruit type—specific	*Fleshy*: Acrosarcum, Amphisarcum, Bacca, Baccarium, Bibacca, Drupe, Pepo, Syconium; *Dry*: Achenarium, Achene, Caryopsis, Ceratium, Craspedium, Cypsela, Denticidial capsule, Disclesium, Fissuricidal capsule, Legume, Loculicidal capsule, Lomentum, Polachenarium, Pyxidium, Samara, Samarium, Septicidial capsule, Utricle	10	1, 11, 18
	Dispersal mode*	Autochory, Anemochory, Hydrochory, Zoochory, or a combination of these	11	18
Extrinsic	Use*	Handicraft, Horticultural, Ornamental, Medicinal, or a combination of these, No use reported	17	18

(1) Acevedo‐Rodriguez, 2005; (2) Axelrod (2011), (3) Carey et al., 2012; (4) Gentry, 1991; (5) Harper, 1977; (6) Más and Lugo‐Torres (2012), (7) Olson et al., 2001; (8) Putz & Mooney, 1991; (9) Richardson et al., 2000; (10) Spjut, 1994; (11) van der Pijl, 1972, (12) Plants USDA database, (13) TROPICOS database, (14) UPR and MAPR Herbaria, (15) E. Santiago (personal observation), (16) D. Delgado (unpublished data), (17) see 2, (18) see Appendix S2.

Traits with (*) were included in the models.

### 2.3 Distribution, abundance, and biogeographic origin

Three complimentary approaches were used to characterize the distribution of vines. First, based on the extent of their distribution, vines were classified into endemic (only recorded in the island), native (recorded in Puerto Rico, the Caribbean, and parts of the Neotropics), casual alien (species not part of the original flora of Puerto Rico that have escaped cultivation), and naturalized alien (species introduced to the island with self‐sustaining populations; Richardson et al., 2000). Second, vines' historic and current biogeographic realms were determined from historic and current occurrences (Appendix S2) in combination with Olson's et al. (2001) map depicting the seven biogeographic realms (Table 1). These biogeographic realms include the Afrotropics (all sub‐Saharan Africa), Australasia (Australia, Papua New Guinea and New Zealand), Indo‐Malay (India, Indonesia and Thailand), Nearctic (North America and Greenland), Neotropics (Central and South America and the Caribbean), and Palearctic (Europe, Asia, and North Africa). Lastly, species were assigned to one of three local abundance categories derived from a semiquantitative measure of abundance derived from Axelrod (2011): common (species found in ≥5 of 14 geographic regions), uncommon (species found in 1–4 geographic regions or ≥3 of 24 designated state forest reserves, or ≥3 of 78 municipalities), and rare (species reported in <3 state forest reserves or <3 municipalities). Thus, this definition of local abundance describes the distribution and not the population size of any given species within the island.

### 2.4 Intrinsic and extrinsic traits

We focused on four intrinsic (life span, climbing mechanism, general and specific fruit type, and dispersal mode) and one extrinsic (plant use) traits that are relevant to understand the proliferation of vines and for which information for most species could be obtained (Table 1; Binggeli, 1996; Thuiller, Richardson, Rouget, Procheş, & Wilson, 2006). Vine species were assigned to either of three life span classes, namely annual, perennial, or annual/perennial (Harper, 1977). The annual/perennial class included species that can act as annuals or perennials depending on climatic or geographic conditions. We also classified vine species according to six climbing mechanism categories (Acevedo‐Rodriguez, 2005; Putz & Mooney, 1991). Three of these (aerial roots, tendrils, twining) involve specialized structures to climb, attach, or twist onto other structures and thus represent active climbing mechanisms. The other three involve habits or structures not necessarily evolved for climbing and thus are referred to as passive climbing mechanisms. These include scandent (species with runners to creep over other structures) and sarmentose (species that can recline and grow over other structures) habits and structures like spines. Vines were also assigned to four general and 25 specific fruit types (Spjut, 1994; Table 1). The general categories included simple (indehiscent fruits derived from one flower and one carpel), schizocarpic (fruits derived from one flower and two or more carpels; instead of dehiscing, fruits split among numerous segments), rhexocarpic (dehiscent fruits derived from one flower and two or more carpels; seeds are shed through sutures or openings of the pericarp), and compound fruits (fruits derived from more than one flower). For 8% of the species, genus level information had to be used. The last intrinsic trait used was seed dispersal mode, with four categories: anemochory (dispersal by wind), hydrochory (dispersal by water), autochory (dispersal by gravity), and zoochory (dispersal by animals, either by endozoochory or exozoochory), or a combination of these deduced from fruit type or from published accounts (van der Pijl, 1972; Appendix S2). Plant use, our extrinsic trait, included four categories: handicraft, ornamental, horticultural, and medicinal or a combination of these if more than one plant use was reported (Table 1). Species classified as having no plant use are those for which none was reported in the literature.

### 2.5 Data analysis

To test for associations between proliferation status and the different categorical variables (family, life form, distribution, abundance, and all the intrinsic and extrinsic traits), we created two‐ and three‐way contingency tables and analyzed them using Fisher's exact tests, chi‐square independence tests with Yate's correction, and log‐linear models (Ott & Longnecker, 2010; Sokal & Rohlf, 1995). We also examined the residuals of the chi‐square tests to identify the cells that made the largest contribution to the results (Ott & Longnecker, 2010). To visualize the nature of the exchange of alien species among biogeographic realms (Table 1), we generated two networks, one for nonproliferating alien and the other for proliferating alien species, using the software Gephi 0.8.2. For each network, we used an input matrix in which the rows and columns depicted the historic and current biogeographic realms of distribution of vines. In these networks, the nodes represent the biogeographic realms and the arrows the exchange among them. The nodes were weighed using the number of species original to each realm and the links were weighed using the number of species that dispersed from their historic realm and colonized other realms.

To predict vine proliferation status, we ran a classification tree analysis and used the subset of traits identified in Table 1. A classification tree partitions the data into groups that reduce the within‐group heterogeneity (McCune & Grace, 2002). The product of this recursive partitioning is a decision tree that predicts species' affiliation to predefined categories—vine proliferation status in our case—based on a group of chosen predictors (Table 1). We used the Gini index to determine each split in the trees so as to increase the homogeneity in each resulting group. To select the best predictive model, we ran a 10‐fold cross‐validation analysis, in which the data are divided into 10 groups of equal size and sequentially one of the groups is excluded from the training data used in the generation of the classification tree. The excluded data are later used to validate the tree (De'ath & Fabricius, 2000). The predictive error of each of the resulting 10 trees is calculated and averaged in order to provide a more realistic prediction error. To select an optimal classification tree, we ran 50 cross‐validations and selected the most frequent (modal) optimal tree selected by the analysis (De'ath & Fabricius, 2000; Pyšek et al., 2012). The quality of the modal tree was measured by calculating the overall percentage of correctly classified species (PCC), the sensitivity (ability to predict proliferation status among proliferating species), and the specificity (ability to predict proliferation status among nonproliferating species) of the tree. We excluded family from the variables examined in our classification tree analysis as a way to reduce redundancy as two of the variables included in the model (i.e., fruit type and plant use) partially reflect the phylogenetic influence. All statistical analyses were done using R version 3.2.3 and the R package *rpart* (Therneau, Atkinson, & Ripley, 2015) was used for the classification tree analysis.

## 3 Results

**Figure 3:**
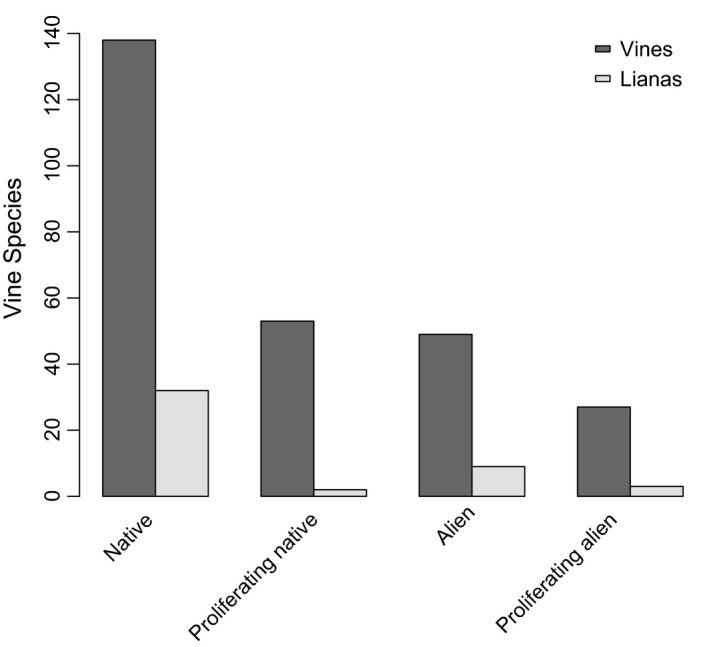
Climbing species classified according to life form, origin, and proliferation status. The proliferating climbing species account for 20% of the native species and 34% of the alien species

Puerto Rico has 313 climbing species, 267 corresponding to vines and the remaining 46 to lianas. A log‐linear model showed that life form, origin, and proliferation status were not independent (*G* ^*2*^ = 13.0, *df* = 4, *p *=* *.01; Figure 3). A subsequent two‐way interaction analysis revealed that the association between life form and proliferation status weighed heavily in this result; specifically, vines were more common among proliferating species, whereas lianas among nonproliferating ones (*G* ^*2*^ = 10.0, *df* = 2, *p *=* *.007). Based on this outcome, we focused the remainder of the study on vines.

**Figure 4:**
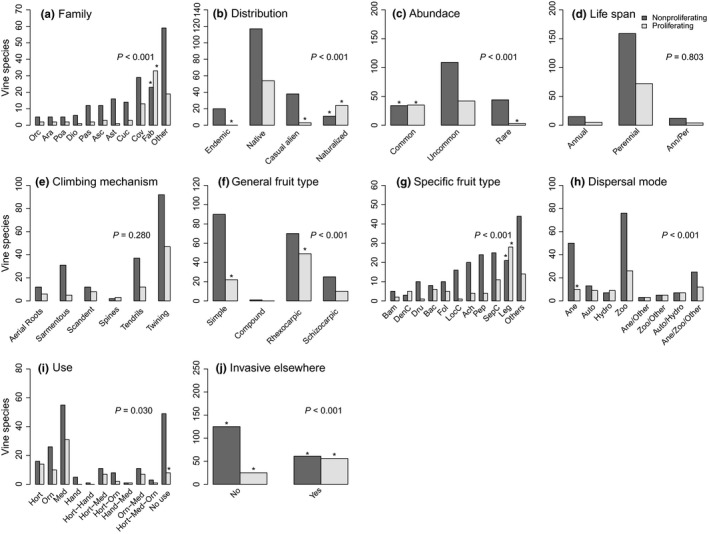
Vine species classified according to intrinsic and extrinsic traits. For each attribute, we show the resulting chi‐square or Fisher's exact test *p*‐value. The asterisks indicate the cells that made the largest contribution to the results. Family: Dio (Dioscoreaceae), Orc (Orchidaceae), Poa (Poaceae), Sap (Sapindaceae), Pas (Passifloraceae), Ast (Asteraceae), Apo (Apocynaceae), Cuc (Cucurbitaceae), Cov (Convolvulaceae), Fab (Fabaceae), Other (32 remaining families), Specific fruit type: FisC (Fissuricidal capsule), Bam (Baccarium), Dru (Drupe), LocC (Loculicidal capsule), Bac (Bacca), Fol (Follicarium), Ach (Achene), Pep (Pepo), SepC (Septicidal capsule), Leg (Legume), and Other (16 remaining fruit types). Dispersal mode: Ane (Anemochory), Auto (Autochory), Hy (Hydrochory)], Zoo (Zoochory). Use: Hand (Handicraft), Hort (Horticultural), Med (Medicinal), and Orn (Ornamental). The y axis varies with the graphs

The 267 species of vines are in 42 families and 126 genera. Five families, namely the Fabaceae, Convolvulaceae, Cucurbitaceae, Apocynaceae, and Asteraceae, contribute 55% of all reported species (Figure 4a). Focusing on species' origin, we found that 28% of the species were alien, and only nine of these alien species belonged to families not previously found in the island. Within the group of alien species, only 10% were proliferating, representing 13 families of which only three are not represented among the native species of the island.

**Figure 5:**
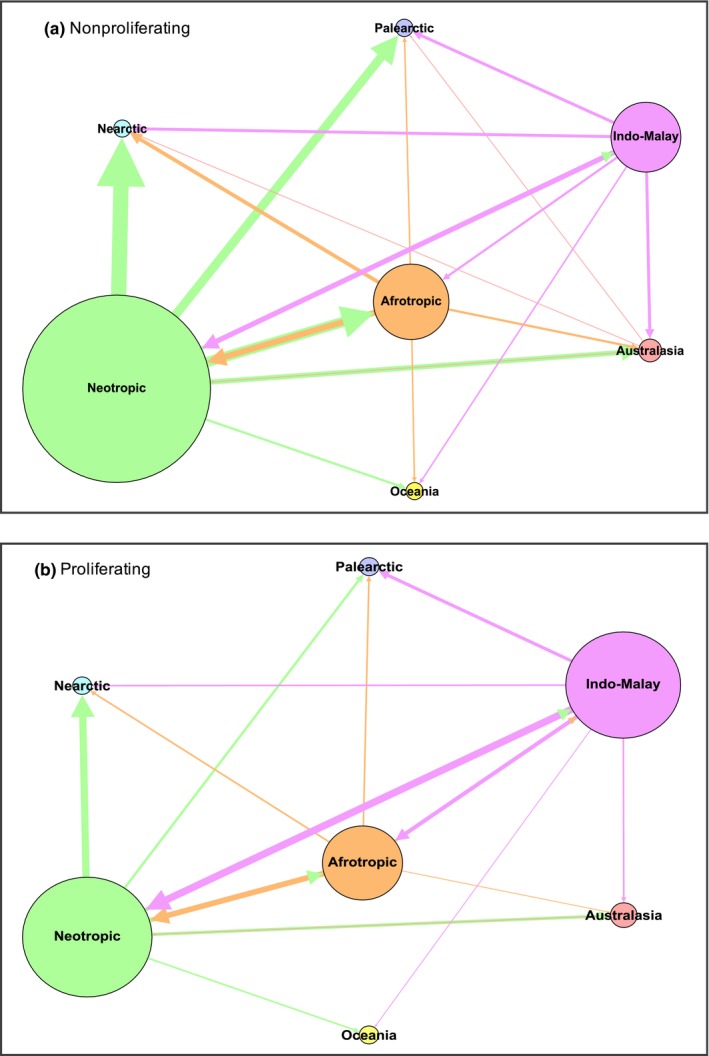
Networks showing the exchange of (a) nonproliferating (*n* = 49) and (b) proliferating (*n* = 27) alien vine species reported for Puerto Rico based on their historic and current biogeographic realms of distribution. In our networks, variation in node size indicates that the realms have made a different contribution to Puerto Rico's subset of alien vines, whereas variation in the number and size of the links indicates the extent of vine exchange among biogeographic realms

The contribution of different biogeographic realms—variation in node size—to the pool of alien vines reported for Puerto Rico, as well as the nature of exchange among them—variation in link number, width, and direction—differed according to proliferation status (Figure 5). The Neotropics followed to a lesser extent by the Indo‐Malay and Afrotropics realms contributed the largest number of nonproliferating, alien species recorded on the Island (Figure 5a). Although these three realms also contributed the largest number of proliferating alien species, the relative importance of the Neotropics diminishes, whereas that of the Indo‐Malay region increases (Figure 5b). Our networks also show that the exchange of nonproliferating vines among biogeographic realms has been greater than for proliferating vines as reflected by differences in link density in the networks. This in part is due to the broad distribution of some nonproliferating alien vines. Finally, realms that are environmentally similar to Puerto Rico contribute more proliferating species than realms that are geographically close.

### 3.1 Vine origin, traits, and proliferation status

Origin of proliferating vines was associated with only one out of seven traits, namely the extrinsic trait plant use (Table 2). We found that more alien, proliferating species than expected by chance were reported to have horticultural and medicinal uses, whereas the opposite was true for native, proliferating ones. These results led us to group together alien and native proliferating species to examine the extent to which proliferation status was associated with the various traits considered in this study.

**Table 2:** Results from Fisher's exact test and chi‐squared tests of independence

Group examined	Trait	χ^2^	*df*	*p*‐value
Native versus alien proliferating species	Local abundance			.15
	Life span			1.00
	Climbing mechanism			.27
	Fruit type (general)			.46
	Fruit type (specific)			.34
	Dispersal mode			.35
	Plant use			**<.01**
Proliferating versus nonproliferating species	Family			**<.01**
	Distribution	47.526	3	**<.01**
	Local abundance	29.674	2	**<.01**
	Life span			.80
	Climbing mechanism			.28
	Fruit type (general)			**<.01**
	Fruit type (specific)			**<.01**
	Dispersal mode			**<.01**
	Plant use			**.03**

Significant values are shown in bold.

In all but two instances, we found an association between proliferation status and the traits included in this study (Table 2; Figure 4). An analysis of the residuals highlighted the categories within each variable that made the largest contribution to the observed results (Figure 4). Our data show that the Fabaceae is the most represented family in the island, and the species within this family contributed more proliferating species than expected by chance (Fisher's exact test *p *<* *.001; Figure 4a). In terms of distribution, the majority of vine species on the island are native, yet there are more proliferating species among the naturalized, than among any of the other classes (χ^2^ = 47.52, *df* = 3, *p *<* *.001; Figure 4b). As expected, there are more proliferating species classified as common species—a group that makes up only 25% of all the vine species in the island—than expected by chance (χ^2^ = 27.0, *df* = 2, *p *<* *.001; Figure 4c). On the other hand, traits such as life span (Fisher's exact test *p *=* *.80; Figure 4d) and climbing mechanism (Fisher's exact test *p *=* *.28, Figure 4e) were independent from proliferation status, with the vast majority of vines being perennial, and twining or tendril climbers.

In terms of fruit type, most Puerto Rican vines produce rhexocarpic and simple fruits (Figure 4f), with more proliferating species producing rhexocarpic fruits than expected by chance (Fisher's exact test, *p *<* *.001). Among the species with rhexocarpic fruits, there were more proliferating species producing legumes than expected by chance (Fisher's exact test, *p *<* *.001; Figure 4g). Vines were characterized by one, two, or more dispersal modes. Overall, zoochory was the prevalent mode of dispersal followed by anemochory (Figure 4h), where anemochory included fewer proliferating species than expected (Fisher's exact test, *p *<* *.01). Seventy‐nine percent of the species had at least one reported plant use (Figure 4i), and among those without a reported plant use, we found fewer proliferating species than expected by chance (Fisher test *p *=* *.03). Finally, proliferation status on the island and invasive status elsewhere were not independent (χ^2^ = 28.81, *df* = 1, *p *<* *.001; Figure 4j). Vine species listed as invasive elsewhere are found more often than expected among proliferating vines in Puerto Rico (Figure 4j). Thus, invasive status elsewhere may provide a good indication of the proliferation potential of a species on the island.

### 3.2 Predicting vine proliferation status

Our classification tree analysis generated trees with an average prediction error of 22%, well below the predicted 50% error of a random classification, and the 31% error of the null model. The majority of the classification trees generated by the 50 cross‐validations had eight leaf nodes, while the optimal selected modal tree was pruned to a size of six leaf nodes in order to obtain a less complex tree with the smallest relative error (Appendix S3). The optimal, modal classification tree had an overall high predictive power (PCC = 82.0%) resulting from its high ability to correctly classify nonproliferating species (Specificity = 92.9%) and its moderate ability to classify proliferating ones (Sensitivity = 57.8%). The optimal, modal tree identified five variables as good predictors of proliferation status and used them to split the species into six groups characterized by a unique combination of traits (Groups in Table 3). Three of these groups included proliferating species, while the other three, nonproliferating ones. The first group of proliferating species included common vines with rhexocarpic fruits (Group 2). The second and third groups included rare and uncommon species. Whereas the second group included naturalized species (Group 3), the third one included casual aliens, native, and endemic species with horticultural and medicinal uses and that have aerial roots and scandent habits (Group 6). The first group of nonproliferating species included common species with simple or schizocarpic fruits (Group 1). The second and third groups of nonproliferating species included rare and uncommon species that are casual aliens, native, or endemic. The second group of nonproliferating species was further distinguished by the presence of vines used mostly for handicraft and ornamental purposes, species that have multiple plant uses (handicraft/horticultural, horticultural/medicinal, medicinal/ornamental) or species with no reported plant use (Group 4). Finally, the third group of nonproliferating vines included species with mostly horticultural and medicinal uses that use spines, tendrils, twining, and sarmentose habits to climb (Group 5).

**Table 3:** Traits and the split conditions identified by the most frequent (modal) optimal classification tree selected from a series of 50‐fold cross‐validations

	Proliferating status	Correctly classified %	Group No.
Local Abundance			
Common			
**Fruit**			
Simple, Schizocarpic	Nonproliferating	69	1
Rhexocarpic	Proliferating	77	2
Rare and uncommon			
**Distribution**			
Naturalized	Proliferating	88	3
Casual alien, Native, and Endemic			
**Plant use**			
Hand, Hand–Hort, Hort–Med, Med–Orn, Orn, No use	Nonproliferating	92	4
Hort, Hort–Orn, Med			
**Climbing mechanism**			
Sarm, Spin, Tend, Twin	Nonproliferating	78	5
AR, Scan	Proliferating	60	6

Plant use: Hand, Handicraft; Hort, Horticultural; Med, Medicinal; Orn, Ornamental.

Climbing mechanism: AR, Aerial Roots; Sarm, Sarmentose; Scan, Scandent; Spin, Spines; Tend, Tendrils; Twin, Twining.

Traits appear in bold.

## 4 Discussion

We proposed to develop an approach that would reduce biases in the prediction of the proliferation status of plants. We did so by leveraging on the diversity of vines and occurrence of alien and native proliferating species on the island of Puerto Rico. As hypothesized, both alien and native proliferating species shared all but one trait, namely plant use. We also established that proliferating and nonproliferating vines differed in all but two of the traits considered in this study, namely life span and climbing mechanism. Finally, we were able to predict vine proliferation status based on five traits, namely local abundance, distribution, general fruit type, climbing mechanism, and plant use.

### 4.1 Distribution, traits and proliferation status

Climatic similarity and geographic distance between the native and invaded range of a species are two known factors that influence the invasive success of alien species. Yet, each factor influences a different stage of the invasion process (Theoarides & Dukes, 2007). Climatic similarity influences the success of the alien species during the colonization and establishment stage of the invasion process. Geographic distance, on the other hand, can act as a major filter during the transport and introduction stage of an invasion, where the probability of propagule transport and introduction decreases with distance (Theoarides & Dukes, 2007). A similar pattern was observed in the pool of alien vines present in Puerto Rico, which makes up 28% of the island's contemporary vine assemblage. First, the majority of the alien species were originally from geographic realms that share a climate similar to that of Puerto Rico (i.e., Neotropic, Indo‐Malay, and Afrotropic). Second, the large portion of nonproliferating vines form the Neotropic that has reached the Nearctic and Palearctic highlights the role of geographic distance facilitating the dispersal of species to closer realms. However, the same pattern is not seen among proliferating alien vines, where climatic similarity, rather than distance, had a stronger influence in their distribution. These results suggest that the successful proliferation of alien species depends greatly on their ability to acclimate to their invaded range.

Further examination of Puerto Rico's contemporary vine assemblage showed major differences between proliferating and nonproliferating species. In terms of taxonomy, two families, the Fabaceae and Convolvulaceae, contribute 56% of all the proliferating species in the island. Two nonmutually exclusive hypotheses may explain these patterns. First, in Puerto Rico the occurrence of native proliferating vines partially reflects the overall richness of these families. Second, the occurrence of alien proliferating vines may reflect the uses given to these plants (see later). In terms of traits, as hypothesized, both intrinsic and extrinsic traits were associated with proliferation status. Two important intrinsic traits, namely fruit type and dispersal mode, reflect various qualities of propagule pressure such as seed number and dispersal distance (Ibáñez et al., 2014; Martínez‐Ghersa & Ghersa, 2006). Our results showed that more proliferating species than expected by chance produced rhexocarpic fruits, whereas more nonproliferating species than expected produced simple fruits. The largest number of species with rhexocarpic fruits is in the Fabaceae and Convolvulaceae, whereas the largest number of species with simple fruits was in the Asteraceae, Cucurbitaceae, and Passifloraceae. These last three families include most of the endemic vines in the island, in particular, the genus *Mikania,* in the Asteraceae, which has the largest number of endemic species in the Caribbean islands (Francisco‐Ortega et al., 2008), accounting for 67% of the endemic species in Puerto Rico. Thus, in the context of our work, fruit type may be an important trait facilitating the proliferation of vines and a useful trait in the discrimination between most proliferating and nonproliferating species.

Plant use, on the other hand, reflects socioeconomic conditions, land‐use patterns, cultural values (Dehnen‐Schmutz et al., 2007; Thuiller et al., 2006), and sowing intensity or propagule pressure (Pyšek et al., 2009), which are all known to favor the introduction and spread of species. In our study, proliferating vines were concentrated in a small number of families and tended to have a reported plant use, especially horticultural and ornamental uses, whereas their nonproliferating counterparts usually were used for handicraft purposes or had no reported plant use. Families with proliferating species (e.g., Fabaceae, Convolvulaceae, Araceae, Cucurbitaceae, and Dioscoreaceae) are widely recognized as economically important due to their horticultural (edible fruits, tubers, cover crops), ornamental, and medicinal value (Daehler, 1998; Gentry, 1991; Langer & Hill, 1991).

### 4.2 Predicting vine proliferation status

The examination of individual traits as wells as the species' global invasive status was useful to better understand the role of these variables in “predisposing” vines to proliferate (Daehler & Carino, 2000; Pheloung, 1995; Pyšek et al., 2009). Yet, it was the classification tree analysis that allowed the identification of vine groups characterized by a unique combination of traits, three of which characterized proliferating vines. In this instance, our work is not the first to use classification trees in combination with plant traits to generate models that predict invasive potential (Reichard & Hamilton, 1997); however, our approach has several advantages. First, the use of trait data that it is easy to gather increases the simplicity and applicability of our approach, especially for regions where knowledge of individual species is limited. Second, our approach focuses in identifying suites of traits, rather than individual traits, that characterize proliferating species, thus allowing us to identify how these traits interact to facilitate vine proliferation. Third, the consideration of both native and alien plant species in our analysis offers a more complete understanding of the traits that facilitate proliferation irrespective of species' origin. Lastly, our classification tree analysis provides a list of species considered false positives, nonproliferating species classified as proliferating by the analysis. These false positives represent species that should be monitored for their potential to proliferate in the future.

Our analysis highlights the importance of the interaction of multiple traits that contribute to the invasive potential of a species. For example, although climbing mechanism as an individual trait was not associated with proliferations status, coupled with distribution and plants use, it does help discriminate between proliferating and nonproliferating species. In particular, one group of proliferating species was composed of vines with horticultural, ornamental, or medicinal uses, and with aerial roots or a scandent climbing mechanism. Root climbers can utilize their specialized adhesive roots to attach to a wide range of natural and artificial substrates (Melzer, Seidel, Strinbrecher, & Speck, 2012), which can facilitate their establishment in a wider range of habitats. Scandent vines, on the other hand, can live as shrubs when trellis is not available (Dey, 2001), which can function as an advantage when colonizing new environments. However, these two climbing mechanisms only seem to facilitate the proliferation of vine species that can be sown in large quantities due to their horticultural value. Some examples of successful proliferating root climber and scadent vines are *Syngonium podophyllum* Schott and *Asystasia gangetica* (L.) T. Anderson, respectively, both species reported as invasive in several parts of the world (Cochrane, 1998; Josekutty, Wakuk, & Joseph, 2002; Space & Flynn, 2000).

The inclusion of native species in our analysis allowed us to examine commonalities between native and alien proliferating species, thus helping us to elucidate which plant traits favor a proliferating behavior in vine species in general. In our analysis, the included 53 native proliferating species accounted for 64% of the total proliferating species in Puerto Rico. These species represented 23 families, nine of them not represented among the alien species in the island. According to our results, two of the described suites of traits characterizing proliferating species describe both native and alien species. These results suggest that native and alien species can share the same suite of traits that facilitate their proliferation. Also, it suggests that the exclusion of native proliferating species from these type of analyses can lead to an incomplete understanding of relevant traits influencing a proliferating behavior.

In addition, our model produced false positives as well as false negatives that deserve further scrutiny. The false positives included 13 species, and seven of these species are considered invasive outside of Puerto Rico (e.g., *Paullinia pinnata* L.; Ndam, Enang, Mih, & Egbe, 2014). Two nonmutually exclusive hypotheses may explain these false positives. First, these species may proliferate in the near future but currently have not had enough time to either escape from cultivation or adapt to the environmental conditions of their new habitat (Harris et al., 2007; Pyšek et al., 2009). This time lag is evident in Puerto Rico where on average proliferating alien species have been present in the island longer than nonproliferating alien ones (Appendix S1). Additionally, this time lag may explain why one of our false positives (i.e., *Thunbergia alata* Bojer ex Sims) has been listed as invasive in Puerto Rico in a recent publication (Rojas‐Sandoval & Acevedo‐Rodriguez, 2015). Second, extrinsic traits not considered here, such as the properties of the invaded landscape, may have limited the ability of these alien species to proliferate (Foxcroft, Pickett, & Cadennasso, 2011; Lonsdale, 1999).

The false negatives, on the other hand, included 35 species. Twenty of these species are listed as invasive in several regions of the World (e.g., *Cryptostegia madagascariensis* Bojer ex Decne, *Mucuna pruriens* (L.) DC, *Ipomoea violacea* L., and *Passiflora foetida* L.; Kairo, Ali, Cheesman, Haysom, & Murphy, 2003; Space & Imada, 2004). Two hypotheses may explain these false negatives, both of then indicative of the limitations of our approach. First, other traits not considered here, such as those reflecting the physiological and demographic requirements of the plants, may help differentiate species within a given family that was incorrectly classified (Gallagher & Leishman, 2012; van Kleunen, Weber, & Fischer, 2010; Lonsdale, 1999; Thuiller et al., 2006). Second, habitat characteristics describing specific edaphic or disturbance conditions may improve the ability of the model to correctly classify the species' proliferation status (Kueffer & Daehler, 2009), but at the expense of creating an increasingly complex model. Also physiological traits, as well as habitat characteristics, are not always easy to acquire for a large group of species, which often makes using them in predictive models impractical, especially for management applications.

### 4.3 Synthesis and application

The proliferation of alien and native species is likely to continue in a world in which multiple, interacting regional and global processes open opportunities for species to move, establish, and proliferate. Thus, the development of tools that can help identify species with a potential to proliferate is critical for management and conservation purposes (Essl et al., 2015). Tools that can minimize biases and make predictions based on easily obtainable trait data are particularly needed in regions with a high taxonomic and functional diversity, and limited ecological knowledge of individual species. In addition, these tools should be capable of incorporating native species since an increasing number of native species are behaving like invasive aliens (Kirkham, 2005; Taylor & Kumar, 2016). We developed a novel approach to predict vine proliferation status that offers these and some additional advantages. First, the tree classification model that we developed allowed us to uncover trait combinations associated with proliferating and nonproliferating species. Second, the errors produced by the model were equally informative because they indicated the need to include additional traits to improve the predictive power of the model or to focus on some species that may proliferate. Lastly, the model can be used to develop dichotomous keys with relatively easy to obtain trait data, and assess the likelihood for alien and native species to proliferate.

Although our study focused on a relatively small region, our approach can be used in other insular and continental tropical settings that may be undergoing similar socioeconomic and land‐use changes as those experienced by Puerto Rico (Yackulic et al., 2011; Figure 2; Appendix S1). According to our model, proliferating vines in Puerto Rican postagricultural landscapes can be grouped according to three different suites of traits. However, the dynamic nature of landscapes in response to changing biophysical and the socioeconomic process is likely to create different conditions that will favor species characterized by different suites of traits. Thus, as regions become more urbanized, the suite of traits favoring the proliferation of plant species in such environments will probably change. The approach developed here has the potential to identify these new suite of traits to help in the assessment of species' proliferating potential.

## Conflict of Interest

None declared.

## Data Accessibility

Appendix S2 is available in the Dryad Digital Repository: http://dx.doi.org/10.5061/dryad.1tj60.
