# Supplementary material for: Using multiple traits to assess the potential of introduced and native vines to proliferate in a tropical region
Source: Ecol Evol. 2016 Nov 21;6(24):8832–45. doi: 10.1002/ece3.2588 (PMC5192952; doi:10.1002/ece3.2588)
Supplement: Supplementary file 1 [file ECE3-6-8832-s001.docx]

**Appendix S1**

**Vine introductions and the forest transition in Puerto Rico**

We examined the introduction of alien vine species to Puerto Rico during the last 136 years by searching the Universidad de Puerto Rico [UPR] and Universidad de Puerto Rico-Mayaguez campus [MAPR] herbaria that have records dating back to the 1880’s. We obtained data on the earliest year of collection for 71 or 93% of the alien vine species and used it to examine differences in the residence time of proliferating and non-proliferating alien vine species. Proliferating species have been present in the island significantly longer than non-proliferating species (82.5 versus 66.6 years; *t* test, *t* = 18.7, df = 69, *P* < 0.001). This clearly shows the existence of a lag time between time of introduction and time to become invasive in the introduced range. We examined the number of introductions with changes in land use over a similar time period. Using data on total area covered by farmland (US Census Bureau 1930, 1940, 1950, 1954, 1974, 1982, 1987, 1997, 2012) and forest (Birdsey and Weaver 1987, Domínguez-Cristobal 2000, Helmer 2004, Parés-Ramos et al. 2008) during the last 136 years we were able to delimit three time periods (see below) characterized by major changes in the expansion and contraction of farmlands and forested areas.

*First period (1880* *-1920)*

The first period is characterized by an increase in farmland area that peaks around 1915, reflecting in part the efforts to increased sugar production. These efforts helped position Puerto Rico among the most important sugar producers of that time (Mintz 1953). During this period the average rate of species introduction was the highest (1 species/year), but most introductions took place between 1915 and 1916 (Fig. 2 of main text). The steep increase in species introductions coincides both with 1) an extensive plant survey done in Puerto Rico and the Virgin Islands - this was the beginning of the *“Scientific Survey”* in the islands (Liogier 1996) and 2) the establishment of the Agricultural Experimental Station which introduced a large number of alien plant species (e.g., Telford and Childers 1947). The latter can partially explain the large number of vine species with multiple uses (horticultural and medicinal mainly) being introduced during that period (Table A1).

*Second period (1921-1960)*

The second period is characterized by a slow but steady reduction on the area covered by farmlands and an increase of forest cover in the island. This reduction trend is influenced by the boom and bust of the agricultural economy of Puerto Rico and a shift to an industrial economy in 1950. In consequence there was a massive farm abandonment and a large part of the population moved from rural areas to the cities (Ayala and Bernabe 2007). During this period the rate of species introduction is reduced to 0.51 species/year and the species being introduced to the island have mostly ornamental uses (Table A1).

*Third period (1961-present)*

The third period is characterized by a marked increase in forested areas, finally exceeding the area covered by farmlands by 2000. This trend is due to the afforestation of previously abandoned farmlands (Grau et al. 2003). During this period the rate of species introduction increases slightly (0.53 species/year) and most of the species being introduced have multiple or horticultural uses (Table A1).

| **Table A1 –** Plant uses of introduced (casual alien and naturalized) vine species according to time since first collection in Puerto Rico. | | | | | |
| --- | --- | --- | --- | --- | --- |
| **Time period** |  | **Single plant use** |  | **Multiple plant uses** | **No plant use** |
|  | Medicinal | Horticultural | Ornamental |  |  |
| 1884-1920 | 6 | 5 | 5 | 24 | 0 |
| 1921-1960 | 0 | 4 | 12 | 4 | 0 |
| 1961-2008 | 1 | 9 | 4 | 9 | 3 |

**References**

Acevedo-Rodriguez, P. 2005. Vines and climbing plants of Puerto Rico and the Virgin Islands. Smithsonian Institution, Washington, D.C., US.

Albright, T. P., D. P. Anderson, N. S. Keuler, S. M. Pearson, and M. G. Turner. 2009. The spatial legacy of introduction: *Celastrus orbiculatus* in the southern Appalachians, USA. Journal of Applied Ecology **46**:1229-1238.

Axelrod, F. S. 2011. A systematic vademecum to the vascular plants of Puerto Rico. Botanical Research Institute of Texas, Forth Worth, Texas, US.

Ayala, C. J., and R. Bernabe. 2007. Puerto Rico in the American century: A history since 1898. The University of North Carolina Press, North Carolina, USA.

Bellard, C., W. Thuiller, B. Leroy, P. Genovesi, M. Bakkenes, and F. Courchamp. 2013. Will climate change promote future invasions? Global Change Biology **19**:3740-3748.

Binggeli, P. 1996. A taxonomic, biogegraphical and ecological overview of invasive woody plants. Journal of Vegetation Science **7**:121-124.

Birdsey, R. A., and P. L. Weaver. 1987. Forest area trends in Puerto Rico. US Department of Agriculture New Orleans, Louisiana, US.

Blaustein, R. J. 2001. Kudzu's invasion into Southern United States life and culture. Pages 55-82 *in* J. A. McNeeley, editor. The great reshuffling: Human dimensions of invasive species. IUCN, Gland, Switzerland.

Bovell-Benjamin, A. C. 2007. Sweet potato: A review of its past, present and future role in human nutrition. Advances in Food and Nutrition Research **52**:1-59.

Carey, M. P., B. L. Sanderson, K. A. Barnas, and J. D. Olden. 2012. Native invaders-challenges for science, management, policy, and society. Frontiers in Ecology and the Environment **10**:373-381.

Clough, Y., H. Faust, and T. Tscharnetke. 2009. Cacao boom and bust: sustainability of agroforests and opportunities for biodiversity conservation. Conservation Letters **2**:197-205.

Cochrane, C. B. 1998. Antibacterial and antifungal screening of Florida's exotic invasive plant species. University of South Florida, Florida, USA.

Daehler, C. C. 1998. The taxonomic distribution of invasive angiosperm plants: Ecological insights and comparison to agricultural weeds. Biological Conservation **84**:167-180.

Daehler, C. C., and D. A. Carino. 2000. Predicting invasive plants: Prospects for a general screeing system based on current regional models. Biological Invasions **2**:92-103.

De'ath, G., and K. E. Fabricius. 2000. Classification and regression rrees: A powerful yet simple technique for ecological data analysis. Ecology **81**:3178-3192.

Dehnen-Schmutz, K., M. Williamson, J. Touza, and C. Perrings. 2007. A century of the ornamental plant trade and its impact on invasion success. Diversity and Distributions **13**:527-534.

Delgado, D. 2015. Disentangling vine-invaded tropical landscapes: From individual patches to vine networks. University of Puerto Rico-Rio Piedras, <http://hdl.handle.net/10586/551>.

Dey, S. C. 2001. Growing shrubs and climbers. Streling paperbacks, New Delhi, India.

Domínguez-Cristobal, C. 2000. Panorama histórico forestal de Puerto Rico. Editorial de la Universidad de Puerto Rico, San Juan, Puerto Rico.

Ernst, J., and P. Ketner. 2007. Study on the ecology and possible control methods of the invasive plant species Antigonon leptopus (Corallita or Mexican Creeper). Corallita Pilot Project, St. Eustatius, Netherlands Antilles - Final Report. Aruba.

Essl, F., S. Bacher, T. M. Blackburn, O. Booy, G. Brundu, S. Brunel, A.-C. Cardoso, R. Eschen, B. Gallardo, B. Galil, E. García-Berthou, P. Genovesi, Q. Groom, C. Harrower, P. E. Hulme, S. Katsanevakis, M. Kenis, I. Kühn, S. Kumschick, A. F. Martinou, W. Nentwig, C. O'Flynn, S. Pagad, J. Pergl, P. Pyšek, W. Rabitsch, D. M. Richardson, A. Roques, H. E. Roy, R. Scalera, S. Schindler, H. Seebens, S. Vanderhoeven, M. Vilà, J. R. U. Wilson, A. Zenetos, and J. M. Jeschke. 2015. Crossing Frontiers in Tackling Pathways of Biological Invasions. Bioscience **65**:769-782.

Ewel, J. J., and J. L. Whitmore. 1973. The Ecological Life Zones of Puerto Rico and the U.S. Virgin Islands. USDA Forest Service, Institute of Tropical Forestry, San Juan, Puerto Rico, US.

Foxcroft, L. C., S. T. A. Pickett, and M. L. Cadennasso. 2011. Expanding the conceptual frameworks of plant invasion ecology. Perspectives in Plant Ecology, Evolution and Systematics **13**:89-100.

Francisco-Ortega, J., I. Ventosa, R. Oviedo, F. Jiménez, P. Herrera, M. Maunder, and J. L. Panero. 2008. Caribbean island Asteraceae: Systematics, molecules, and conservation on a biodiversity hotspot. The Botanical Review **2008**:112-131.

Gallagher, R. V., and M. R. Leishman. 2012. A global analysis of trait variation and evolution in climbing plants. Journal of Biogeography **39**:1757-1771.

Gentry, A. H. 1991. The distribuiton and evolution of climbing plants. Pages 3-49 *in* F. E. Putz and H. A. Mooney, editors. The biology of vines. Cambridge Universtiy Press, Cambridge, UK.

Grau, H. R., T. M. Aide, J. K. Zimmerman, J. R. Thomlinson, E. Helmer, and X. Zou. 2003. The ecological consequences of socioeconomic and land-use changes in postagriculture Puerto Rico. Bioscience **53**:1159-1159.

Grau, H. R., M. E. Hernandez, J. Gutierrez, N. I. Gasparri, M. C. Casavecchia, E. E. Flores-Ivaldi, and L. Paolini. 2008. A peri-urban Neotropical forest transition and its consequences for environmental services. Ecology and Society **13**:35. [online] URL: <http://www.ecologyandsociety.org/vol13/iss31/art35/>.

Ha, D. T., and G. Shively. 2008. Coffee boom, voffee bust and smallholder response in Vietnam's highlands. Review of Develpment Economics **12**:312-326.

Hairiah, K., and M. van Noordwijk. 1989. Root distribution of leguminous cover crops in the humid tropics and effects on a subsequent maize crop. Pages 157-169 *in* J. van der Heide, editor. Nutrient management for food crop production in tropical farming systems. Institute for Soil Fertility and University of Brawijaya, Haren, The Netherlands and Maland, Indonesia.

Harper, J. L. 1977. Population biology of plants. Academic Press, San Diego, California, US.

Harris, C., B. R. Murray, G. C. Hose, and M. A. Hamilton. 2007. Introduction history and invasion success in exotic vines introduced to Australia. Diversity and Distributions **13**:467-475.

Hegarty, E. E., and G. Caballé. 1991. Distribution and abundance of vines in forest communities. Pages 313-335 *in* F. E. Putz and H. A. Mooney, editors. The Biology of Vines. Cambridge University Press, New York, New York, US.

Helmer, E. 2004. Forest conservation and land development in Puerto Rico. Landscape Ecology **19**:29-40.

Helmer, E. H., T. J. Brandeis, A. E. Lugo, and T. Kennaway. 2008. Factors infuencing spatial patterns in tropical forest clearance and stand age: Implications for carbon storage and species diversity. Journal of Geophysical Research **113**:G02S04, doi:10.1029/2007JG000568.

Higgins, S. I., D. M. Richardson, and R. M. Cowling. 1996. Modeling Invasive Plant Spread: The Role of Plant-Environment Interactions and Model Structure. Ecology **77**:2043-2054.

Huang, Q. Q., J. M. Wu, Y. Y. Bai, L. Zhou, and G. X. Wang. 2009. Identifying the most noxious invasive plants in China: Role of geographical origin, life form and means of introduction. Biodiversity and Conservation **18**:305-316.

Ibáñez, I., J. M. Diez, L. P. Miller, J. D. Olden, C. J. B. Sorte, D. M. Blumenthal, B. A. Bradley, C. M. D’Antonio, J. S. Dukes, R. I. Early, E. D. Grosholz, and J. J. Lawler. 2014. Integrated assessment of biological invasions. Ecological Applications **24**:25-37.

Johnson, V. A., J. A. Litvaitis, T. D. Lee, and S. D. Frey. 2006. The role of spatial and temporal scale in colonization and spread of invasive shrubs in early successional habitats. Forest Ecology and Management **228**:124-134.

Josekutty, P. C., E. E. Wakuk, and M. J. Joseph. 2002. Invasive/weedy angiosperms in Kosrae, Federated States of Micronesia. Micronesica Supplement **6**:61-65.

Kairo, M., B. Ali, O. Cheesman, K. Haysom, and S. Murphy. 2003. Invasive species threats in the Caribbean region. Report to the Nature Conservacy. CAB International and CABI Bioscience, Trinidad & Tobago, West Indies.

Kirkham, W. S. 2005. Valuing Invasives: Understanding the *Merremia peltata* invasion in post-colonial Samoa. Ph. D. University of Texas at Austin, Austin, Texas, US.

Knapp, P. A., and P. T. Soulé. 1998. Recent Juniperus occidentalis (Western Juniper) expasion on a protected site in central Oregon. Global Change Biology **4**:357-367.

Kolawole, G. O., and B. T. Kang. 1997. Effect of seed size and phosphorus fertilization on growth of selected legumes. Communications in Soil Science and Plant Analysis **28**:1223-1235.

Kueffer, C., and C. C. Daehler. 2009. A habit-classification framework and typology for understanding, valuing, and managing invasive species impacts. Pages 261-277 *in* S. Inderjit, editor. Management of invasive weeds. Springer, Houten, The Netherlands.

Ladwig, L. M., and S. J. Meiners. 2009. Impacts of temperate lianas on tree growth in young deciduous forests. Forest Ecology and Management **259**:195-200.

Lambert, J. D. H., and J. T. Arnason. 1986. Nutrient dynamics in milpa agriculture and the role of weeds in initial stages of secondary succession in Belize, C. A. Plant and Soil **93**:303-322.

Langer, R. H. M., and G. D. Hill. 1991. Agricultural plants. Cambridge University Press, New York, New York, US.

Liengola, I. 2008. Impact of the invasive liana Sericostachys scandens on forest composition: Implications for the recovery of Grauer's Gorilla in th Kahuzi-Biega National Park, Democratic Republic of Congo. Tropical Resources Bulleting **27**:43-50.

Liogier, H. A. 1996. Botany and botanist in Puerto Rico. Annals of the New York Academy of Scieces **776**:41-53.

Lonsdale, W. M. 1999. Global patterns of plant invasions and the concept of invasibility. Ecology **80**:1522-1536.

Mackey, A. P., K. Carsten, P. Jamaes, N. March, N. Noble, B. Palmer, J. Vitelli, and M. Vitelli. 1996. Rubber vine (*Cryptostegia grandiflora*) in Queensland. Queensland Goverment Natural Resources and Mines, Brisbane, Australia.

Martínez-Ghersa, M. A., and C. M. Ghersa. 2006. The relationship of propagule pressure to invasion potential in plants. Euphytica **148**:87-96.

Más, E. G., and M. d. L. Lugo-Torres. 2013. Malezas Comunes en Puerto Rico e Islas Virgenes Americanas/Common Weeds in Puerto Rico and the US Virgin Islands. University of Puerto Rico, Recinto Universitario de Mayagüez/Mayagüez Campus, USDA Servicio de Conservación de Recursos Naturales Area del Caribe/Natural Resources Conservation Service Caribbean Area, Mayagüez, Puerto Rico.

McCune, B., and J. B. Grace. 2002. Analysis of ecological communities. MjM Software Design, Gleneden Beach, Oregon, US.

Melzer, B., R. Seidel, T. Strinbrecher, and T. Speck. 2012. Structure, attachment properties, and ecological importance of the attachment system of English ivy (*Hedera helix*). Journal of Experimental Botany **62**:191-201.

Meyerson, J. E., and H. A. Mooney. 2007. Invasive alien species in an era of globalization. Frontiers in Ecology and the Environment **5**:199-208.

Mintz, S. W. 1953. The culture history of a Plantation: 1876-1949. The Hispanic American Historical Review **33**:224-251.

Ndam, L. M., J. E. Enang, A. M. Mih, and E. A. Egbe. 2014. Weed diversity in maize (Zea mays L.) fields in South Western Cameroon. International Journal of Current Microbiology and applied sciences **3**:173-180.

Nicodemo, M. L. F., F. H. D. De Souza, J. R. M. Pezzopane, J. C. T. Mendes, W. Barioni, P. Tholon, and P. M. Santos. 2015. Performance of tropical legumes grown as understory of a eucalypt plantation in a seasonally dry area of the Brazilian Cerrado. Tropical grasslands **3**:151-160.

Olson, D. M., E. Dinerstein, E. D. Wikramanaya, N. D. Burgess, G. V. N. Powell, E. C. Underwood, J. A. D. Amico, I. Itoua, H. E. Strand, J. C. Morrison, C. J. Loucks, T. F. Allnutt, T. H. Ricketts, Y. Kura, J. F. Lamoreux, W. W. Wettengel, P. Hedao, and K. R. Kassem. 2001. Terrestrial ecoregions of the World: A new map of Life on Earth. Bioscience **51**:933-938.

Ortiz-Ceballos, A. I., J. R. Aguirre-Rivera, M. M. Osorio-Arce, and C. Peña-Valdivia. 2012. Velvet Bean (*Mucuna pruriens* var. utilis) a cover crop as bioherbicide to preserve the environmental

services of soil. Page 248 *in* R. Alvarez-Fernandez, editor. Herbicides - Environmental impact studies and management approaches. In Tech, Rijeka, Croatia

Ott, L. R., and M. T. Longnecker. 2010. An introduction to statistical methods and data analysis. Brooks/Cole, Belmont, California, US.

Parés-Ramos, I. K., W. A. Gould, and T. M. Aide. 2008. Agricultural abandonment, suburban growth, and forest expansion in Puerto Rico between 1991 and 2000. Ecology and Society **13**.

Perrings, C., H. A. Mooney, and M. Williamson. 2010. The problem of biological invasions. Pages 1-18 *in* C. Perrings, H. A. Mooney, and M. Williamson, editors. Bioinvasions and Globalization—Ecology, Economics, Management, and Policy. Oxford University Press, New York, USA.

Pheloung, P. C. 1995. Determining the weed potential of new plant introductions to Australia. Australia Weeds Committee and the Plant Industries Committee, Perth, Australia.

Putz, F. E., and H. A. Mooney. 1991. The biology of vines. Cambridge University Press, Cambridge, UK.

Pyšek, P., V. Jarošík, P. E. Hulme, J. Pergl, M. Hejda, U. Schaffner, and M. Vilá. 2012. A global assessment of invasive plant impacts on resident species, communities and ecosystems: the interaction of impact measures, invading species’ traits and environment. Global Change Biology **18**:1725-1737.

Pyšek, P., M. Křivánek, and V. Jarošík. 2009. Planting intensity, residence time, and species traits determine invasion success of alien woody species. Ecology **90**:2734-2744.

Reichard, S. H., and C. W. Hamilton. 1997. Predicting invasions of woody plants introduced into North America. Conservation Biology **11**:193-203.

Reichard, S. H., and P. White. 2001. Horticulture as a pathway of invasive plant introductions in the United States. Bioscience **51**:103-113.

Richardson, D. M., P. Pyšek, M. Rejmánek, M. G. Barbour, F. D. Panetta, and C. J. West. 2000. Naturalization and invasion of alien plants: Concepts and definitions. Diversity and Distributions **6**:93-107.

Rojas-Sandoval, J., and P. Acevedo-Rodriguez. 2015. Naturaization and invasion of alien plants in Puerto Rico and the Virgin Islands. Biological Invasions **17**:149-163.

Rosenberry, W., L. Gudmundson, and M. Samper. 1995. Coffee, society, and power in Latin America. John Hopkins University Press, Baltimore, Baltimore, US.

Seebens, H., F. Essl, W. Dawson, N. Fuentes, D. Moser, J. Pergl, P. Pyšek, M. van Kleunen, E. Weber, M. Winter, and B. Blasius. 2015. Global trade will accelerate plant invasions in emerging economies under climate change. Global Change Biology:n/a-n/a.

Simberloff, D. 2011. Native Invaders. Pages 472-475 *in* D. Simberloff and M. Rejmanek, editors. Encyclopedia of Biological Invasions. University of California Press, Berkeley and Los Angeles, California, US.

Sokal, R. R., and F. J. Rohlf. 1995. Biometry. W. H. Freeman and Company, New York, New York, US.

Space, J. C., and T. Flynn. 2000. Observations on invasive plant species in American Samoa. USDA Forest Service, Institute of Pacific Island Forestry, Honolulu, HI, USA.

Space, J. C., and C. T. Imada. 2004. Report to the Republic of Kiribati on invasive plant species on the Islands of Tarawa, Abemama, Butaritari and Maiana. U.S. Forest Service Institute of Pacific Forestry and Bishop Museum Pacific Biological Survey, Honolulu, Hawai'i, US.

Spjut, R. W. 1994. A systematic treatment of fruit types. Memoirs of the New York Botanical Garden **70**.

Standish, R. J., V. A. Cramer, and R. J. Hobbs. 2008. Land-use legacy and the persistence of invasive *Avena barbata* on abandoned farmland. Journal of Applied Ecology **45**:1576-1583.

Taylor, S., and L. Kumar. 2016. Will climate change impact the potential distribution of a native vine (Merremia peltata) which is behaving invasively in the Pacific region? Ecology and Evolution **6**:742-754.

Telford, E. A., and N. F. Childers. 1947. Tropical Kudzu in Puerto Rico. USDA Federal Experimental Station in Puerto Rico Circular **27**:1-29.

Theoarides, K. A., and J. S. Dukes. 2007. Plant invasion across space and time: factors affecting nonindigenous species success during four stages of invasion. New Phytologist **176**:256-273.

Therneau, T., B. Atkinson, and B. Ripley. 2015. rpart: Recursive partition and regression trees. [https://cran.r-project.org/package=rpart](https://CRAN.R-project.org/package=rpart).

Thuiller, W., D. M. Richardson, M. Rouget, Ş. Procheş, and J. R. U. Wilson. 2006. Interactions between environment, species traits, and human uses describe patterns of plant invasions. Ecology **87**:1755-1769.

US Census Bureau. 1930. Fifteenth census of the United States: 1930. Washington, D.C.

US Census Bureau. 1940. The sixteenth census of the United States: 1940. Washington, DC.

US Census Bureau. 1950. Census of Population: 1950 Washington, DC.

US Census Bureau. 1954. United States census of agriculture: 1954. Washington, US.

US Census Bureau. 1974. Census of agriculture: 1974. Washington, US.

US Census Bureau. 1982. Census of agriculture: 1982. Washington, US.

US Census Bureau. 1987. Census of agriculture: 1987. Washington, US.

US Census Bureau. 1997. Census of agriculture: 1997. Washington, US.

US Census Bureau. 2012. Census of agriculture: 2012. Washington, US.

van der Pijl, L. 1972. Principles of dispersal in higher plants. Springer- Verlag, Berlin, Germany.

van Kleunen, M., E. Weber, and M. Fischer. 2010. A meta-analysis of trait differenes between invasive and non-invasive plant species. Ecology Letters **13**:235-245.

Wilson, J. R. U., C. Gairifo, M. R. Gibson, M. Arianoutsou, B. B. Bakar, S. Baret, L. Celesti-Grapow, J. M. DiTomaso, J.-M. Dufour-Dror, C. Kueffer, C. A. Kull, J. H. Hoffmann, F. A. C. Impson, L. L. Loope, E. Marchante, H. Marchante, J. L. Moore, D. J. Murphy, J. Tassin, A. Witt, R. D. Zenni, and D. M. Richardson. 2011. Risk assessment, eradication, and biological control: global efforts to limit Australian acacia invasions. Diversity and Distributions **17**:1030-1046.

Yackulic, C. B., M. Fagan, M. Jain, A. Jina, Y. Lim, M. Marlier, R. Muscarella, P. Adame, R. De Fries, and M. Uriarte. 2011. Biophysical and socioeconomic factors associated with forest transitions at multiple spatial and temporal scales. Ecology and Society **16**:15.
