# Supplementary material for: Using multiple traits to assess the potential of introduced and native vines to proliferate in a tropical region
Source: Ecol Evol. 2016 Nov 21;6(24):8832–45. doi: 10.1002/ece3.2588 (PMC5192952; doi:10.1002/ece3.2588)

**Appendix S3**

**Results from the Classification Tree Analysis used to predict vine proliferation status from intrinsic and extrinsic traits**

**Figure A2.** Results from the 50 cross validation process for the selection of the optimal classification tree. Grey bars show the number of trees of different sizes generated in the process. The line represents the most frequently generated (modal) tree, where the dots show the mean relative error of the tree at each tree size and the error bars represent the standard deviation.


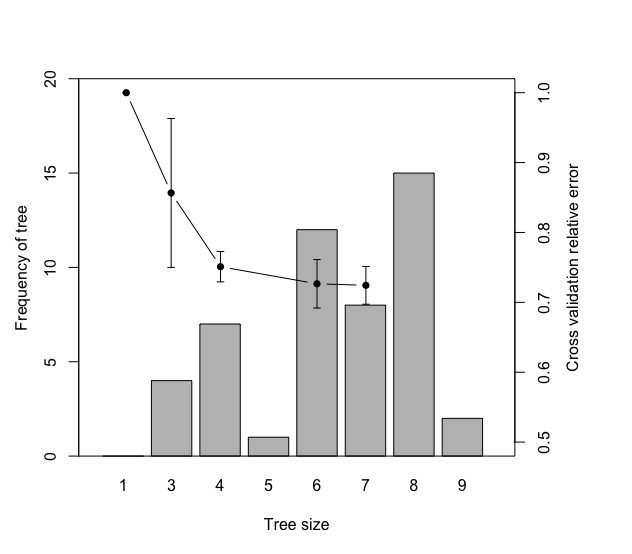

Supplement: Supplementary file 2 [file ECE3-6-8832-s002.docx]
